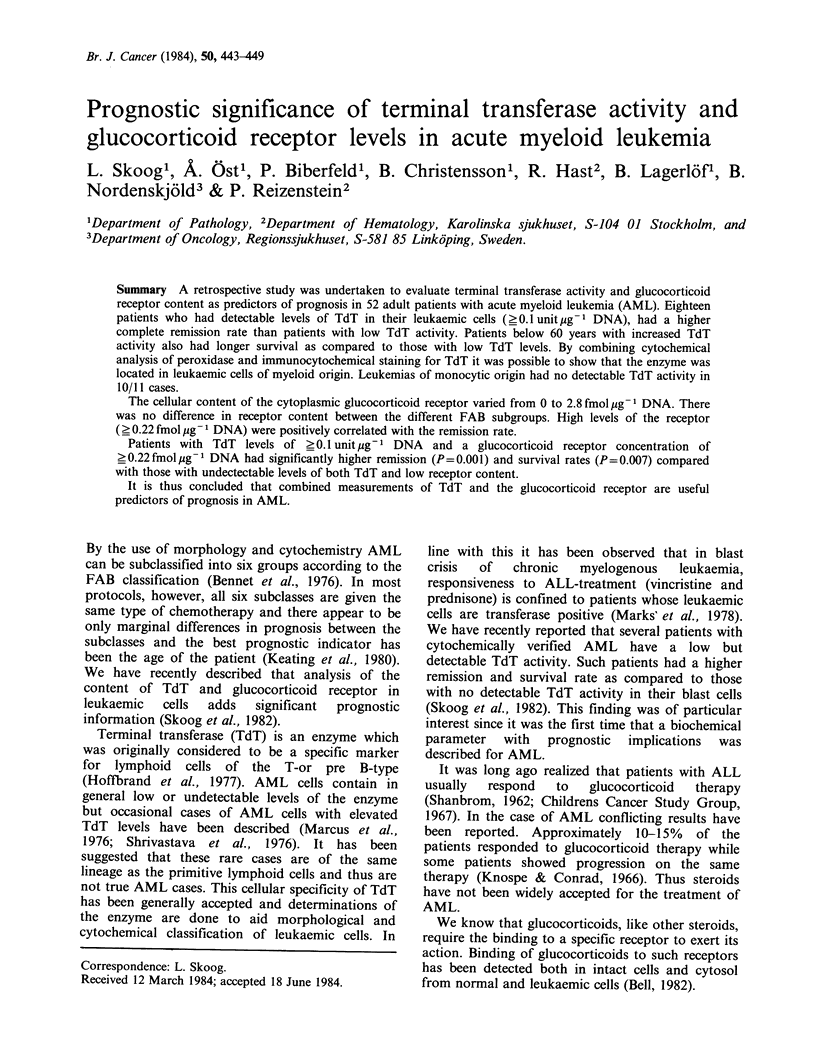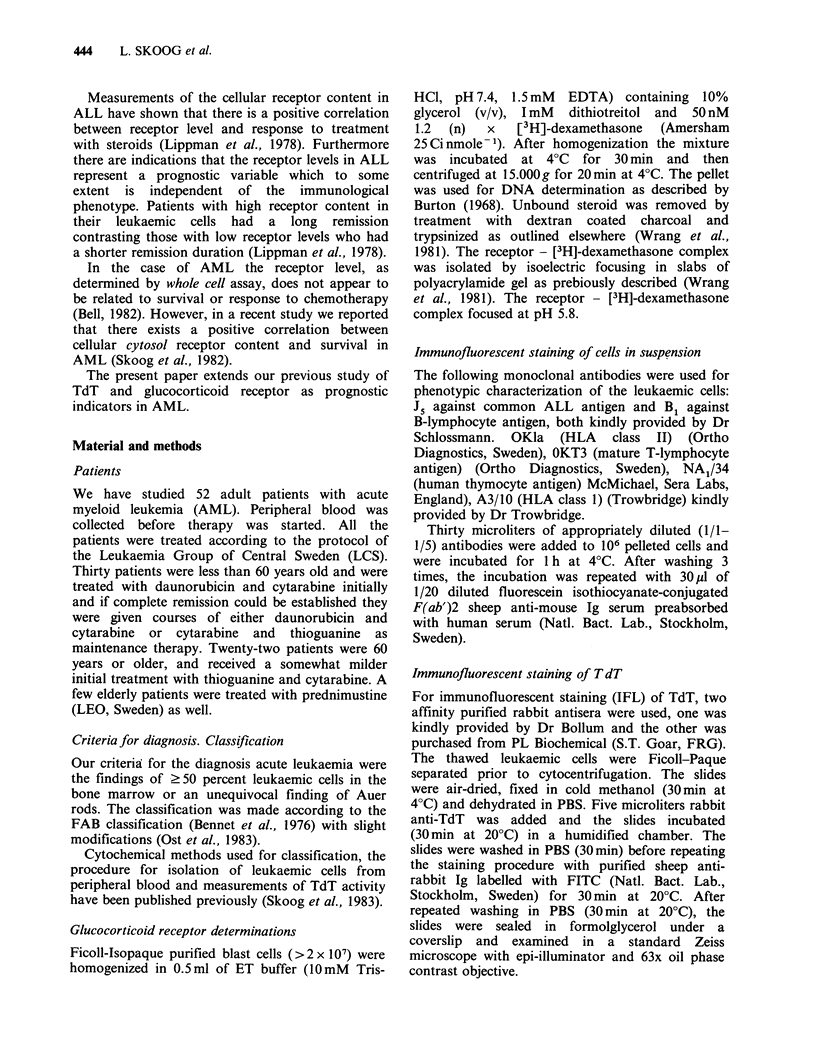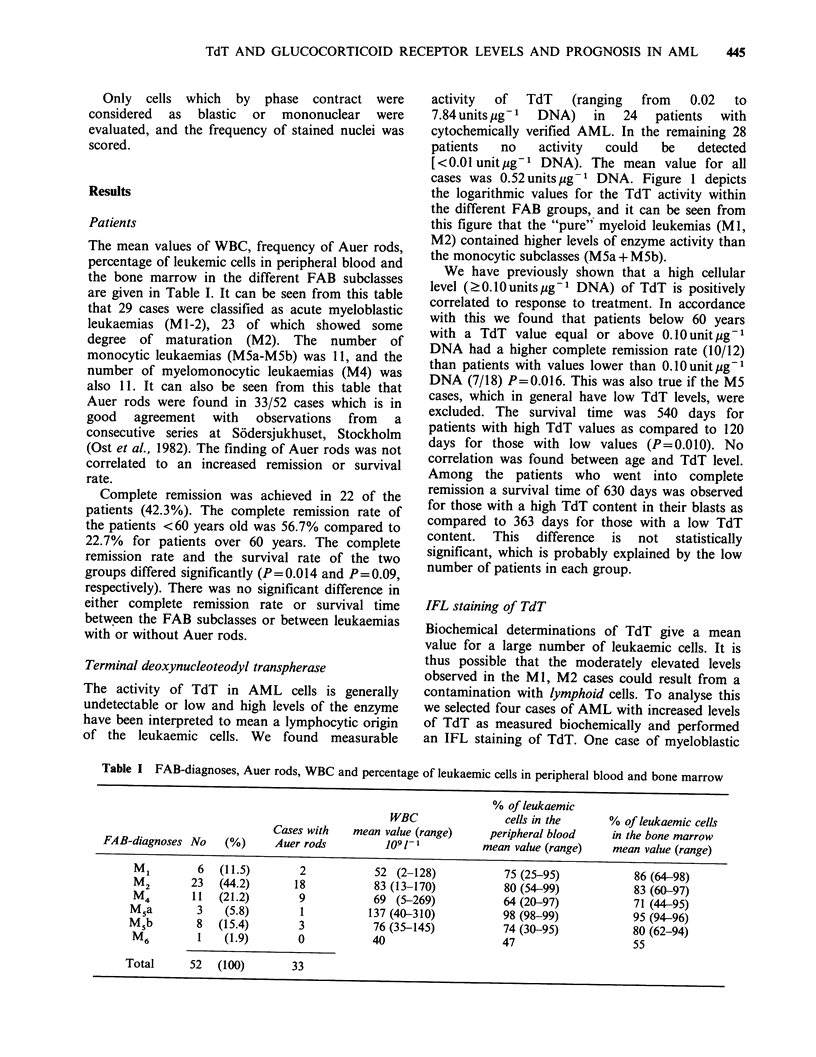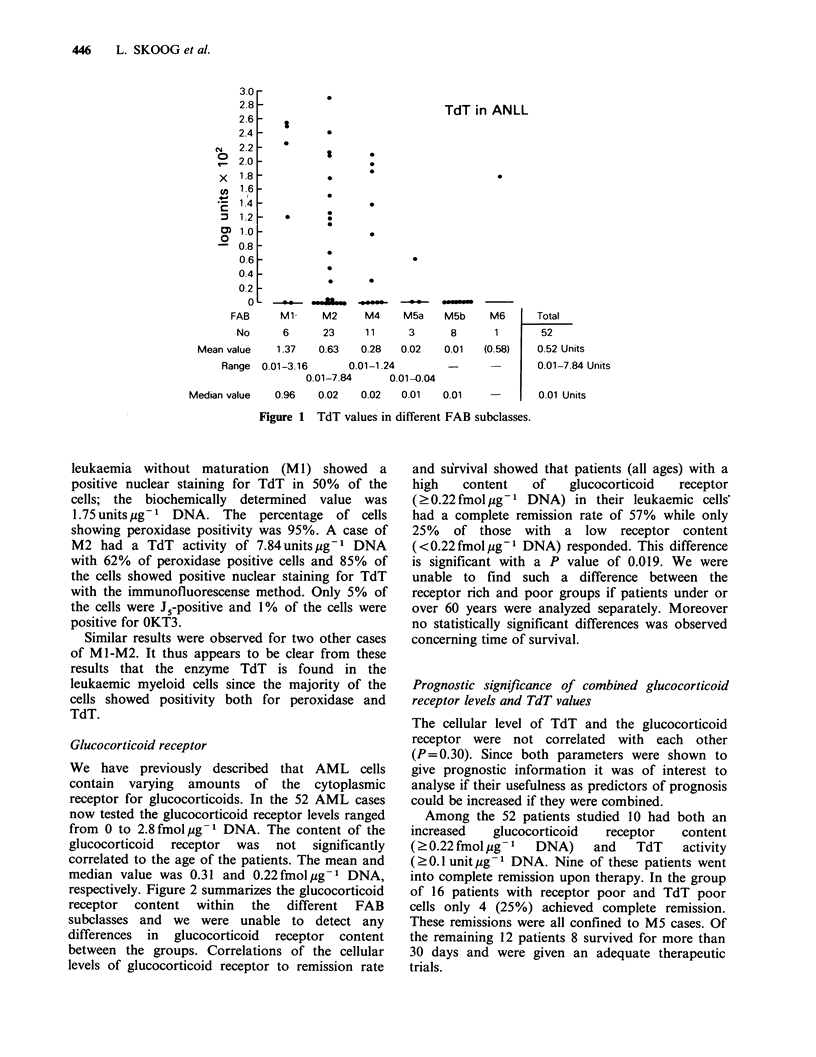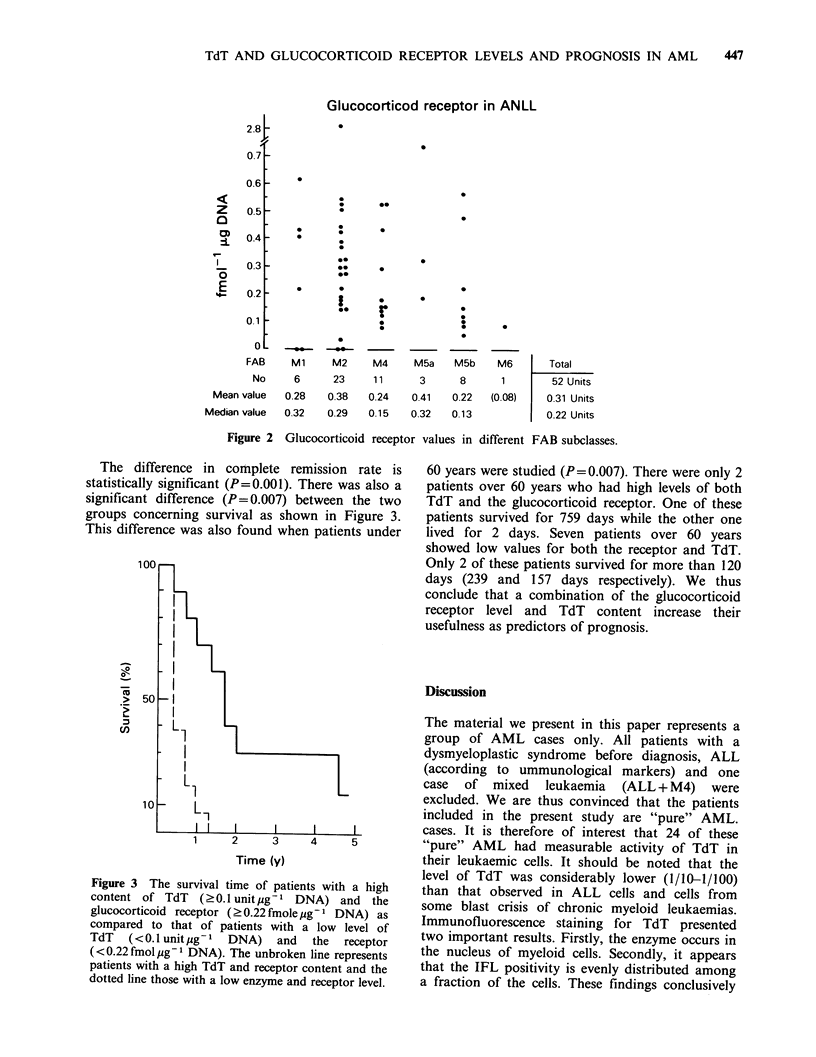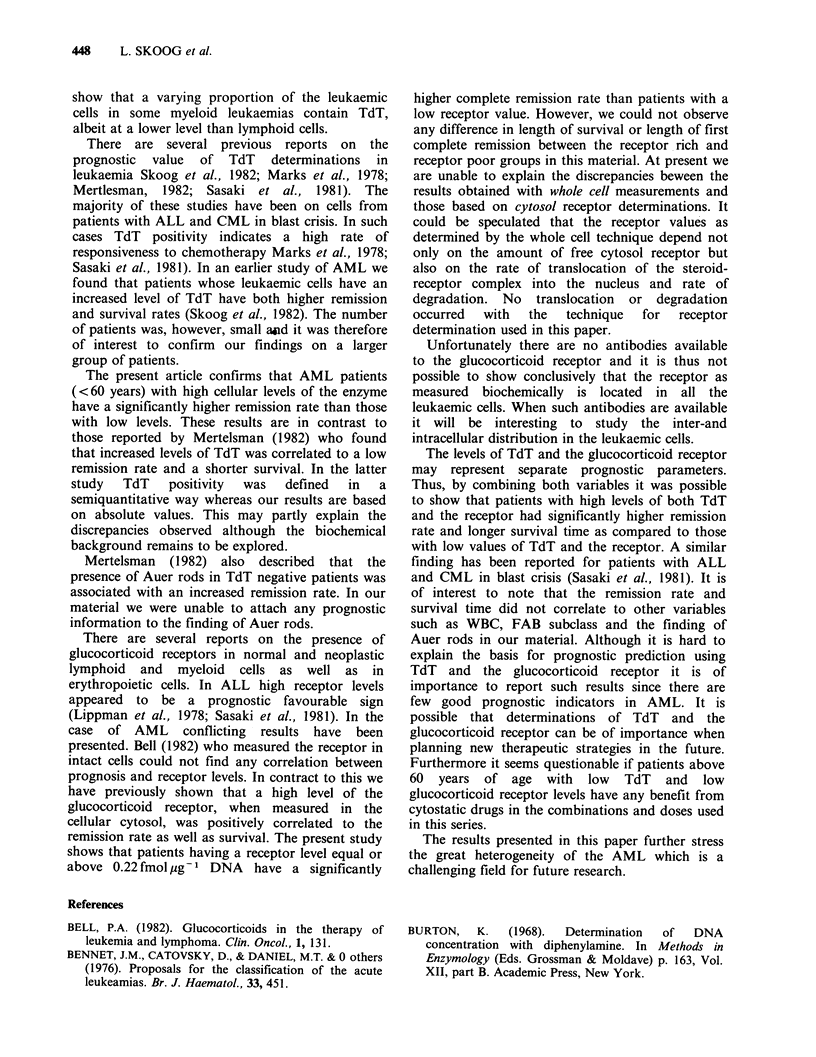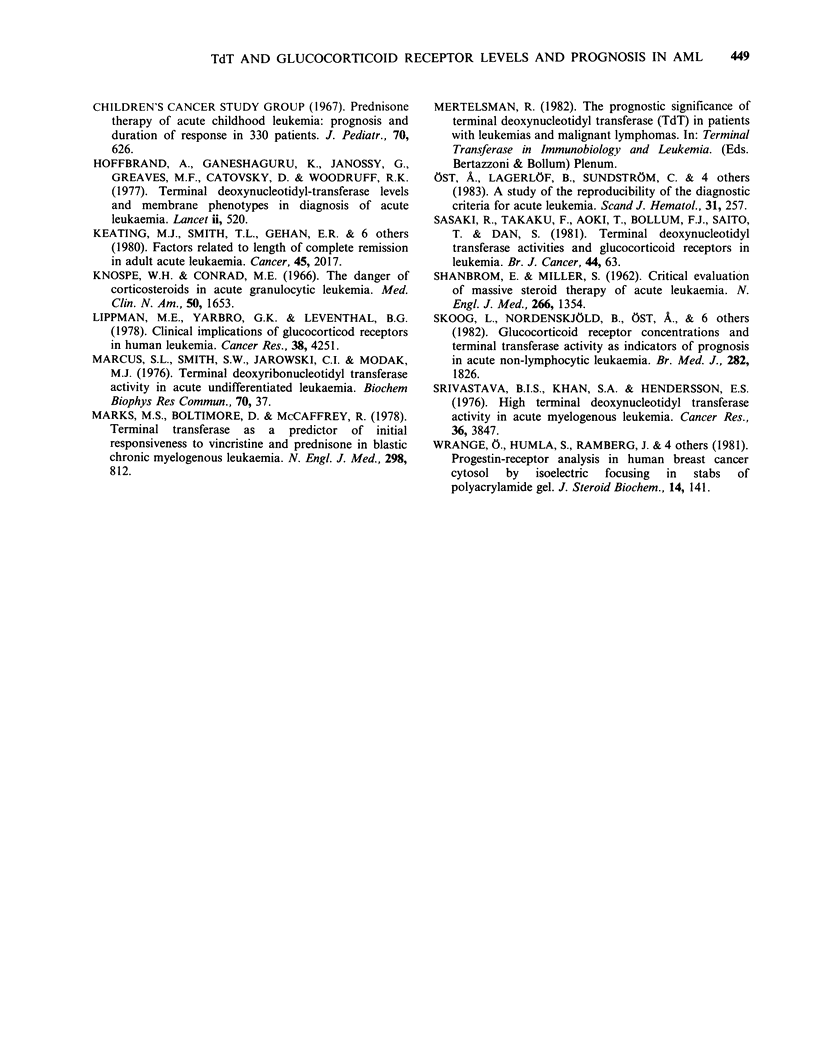# Prognostic significance of terminal transferase activity and glucocorticoid receptor levels in acute myeloid leukemia.

**DOI:** 10.1038/bjc.1984.199

**Published:** 1984-10

**Authors:** L. Skoog, A. Ost, P. Biberfeld, B. Christensson, R. Hast, B. Lagerlöf, B. Nordenskjöld, P. Reizenstein

## Abstract

A retrospective study was undertaken to evaluate terminal transferase activity and glucocorticoid receptor content as predictors of prognosis in 52 adult patients with acute myeloid leukemia (AML). Eighteen patients who had detectable levels of TdT in their leukaemic cells (greater than or equal to 0.1 unit microgram-1 DNA), had a higher complete remission rate than patients with low TdT activity. Patients below 60 years with increased TdT activity also had longer survival as compared to those with low TdT levels. By combining cytochemical analysis of peroxidase and immunocytochemical staining for TdT it was possible to show that the enzyme was located in leukaemic cells of myeloid origin. Leukemias of monocytic origin had no detectable TdT activity in 10/11 cases. The cellular content of the cytoplasmic glucocorticoid receptor varied from 0 to 2.8 fmol micrograms-1 DNA. There was no difference in receptor content between the different FAB subgroups. High levels of the receptor (greater than or equal to 0.22 fmol microgram-1 DNA) were positively correlated with the remission rate. Patients with TdT levels of greater than or equal to 0.1 unit microgram-1 DNA and a glucocorticoid receptor concentration of greater than or equal to 0.22 fmol microgram-1 DNA had significantly higher remission (P = 0.001) and survival rates (P = 0.007) compared with those with undectectable levels of both TdT and low receptor content. It is thus concluded that combined measurements of TdT and the glucocorticoid receptor are useful predictors of prognosis in AML.


*Br. J. Cancer* (1984), **50**, 443–449

### Prognostic significance of terminal transferase activity and glucocorticoid receptor levels in acute myeloid leukemia

L. Skoog[1], Å. Öst[1], P. Biberfeld[1], B. Christensson[1], R. Hast[2], B. Lagerlöf[1], B. Nordenskjöld[3] & P. Reizenstein[2]

[1]*Department of Pathology,* [2]*Department of Hematology, Karolinska sjukhuset, S-104 01 Stockholm, and* [3]*Department of Oncology, Regionssjukhuset, S-581 85 Linköping, Sweden.*

**Summary** A retrospective study was undertaken to evaluate terminal transferase activity and glucocorticoid receptor content as predictors of prognosis in 52 adult patients with acute myeloid leukemia (AML). Eighteen patients who had detectable levels of TdT in their leukaemic cells ($\geq$0.1 unit $\mu g^{-1}$ DNA), had a higher complete remission rate than patients with low TdT activity. Patients below 60 years with increased TdT activity also had longer survival as compared to those with low TdT levels. By combining cytochemical analysis of peroxidase and immunocytochemical staining for TdT it was possible to show that the enzyme was located in leukaemic cells of myeloid origin. Leukemias of monocytic origin had no detectable TdT activity in 10/11 cases.

The cellular content of the cytoplasmic glucocorticoid receptor varied from 0 to 2.8 fmol $\mu g^{-1}$ DNA. There was no difference in receptor content between the different FAB subgroups. High levels of the receptor ($\geq$0.22 fmol $\mu g^{-1}$ DNA) were positively correlated with the remission rate.

Patients with TdT levels of $\geq$0.1 unit $\mu g^{-1}$ DNA and a glucocorticoid receptor concentration of $\geq$0.22 fmol $\mu g^{-1}$ DNA had significantly higher remission ($P$=0.001) and survival rates ($P$=0.007) compared with those with undectectable levels of both TdT and low receptor content.

It is thus concluded that combined measurements of TdT and the glucocorticoid receptor are useful predictors of prognosis in AML.

By the use of morphology and cytochemistry AML can be subclassified into six groups according to the FAB classification (Bennet *et al.*, 1976). In most protocols, however, all six subclasses are given the same type of chemotherapy and there appear to be only marginal differences in prognosis between the subclasses and the best prognostic indicator has been the age of the patient (Keating *et al.*, 1980). We have recently described that analysis of the content of TdT and glucocorticoid receptor in leukaemic cells adds significant prognostic information (Skoog *et al.*, 1982).

Terminal transferase (TdT) is an enzyme which was originally considered to be a specific marker for lymphoid cells of the T-or pre B-type (Hoffbrand *et al.*, 1977). AML cells contain in general low or undetectable levels of the enzyme but occasional cases of AML cells with elevated TdT levels have been described (Marcus *et al.*, 1976; Shrivastava *et al.*, 1976). It has been suggested that these rare cases are of the same lineage as the primitive lymphoid cells and thus are not true AML cases. This cellular specificity of TdT has been generally accepted and determinations of the enzyme are done to aid morphological and cytochemical classification of leukaemic cells. In line with this it has been observed that in blast crisis of chronic myelogenous leukaemia, responsiveness to ALL-treatment (vincristine and prednisone) is confined to patients whose leukaemic cells are transferase positive (Marks' *et al.*, 1978). We have recently reported that several patients with cytochemically verified AML have a low but detectable TdT activity. Such patients had a higher remission and survival rate as compared to those with no detectable TdT activity in their blast cells (Skoog *et al.*, 1982). This finding was of particular interest since it was the first time that a biochemical parameter with prognostic implications was described for AML.

It was long ago realized that patients with ALL usually respond to glucocorticoid therapy (Shanbrom, 1962; Childrens Cancer Study Group, 1967). In the case of AML conflicting results have been reported. Approximately 10–15% of the patients responded to glucocorticoid therapy while some patients showed progression on the same therapy (Knospe & Conrad, 1966). Thus steroids have not been widely accepted for the treatment of AML.

We know that glucocorticoids, like other steroids, require the binding to a specific receptor to exert its action. Binding of glucocorticoids to such receptors has been detected both in intact cells and cytosol from normal and leukaemic cells (Bell, 1982).

Correspondence: L. Skoog.
Received 12 March 1984; accepted 18 June 1984.

Measurements of the cellular receptor content in ALL have shown that there is a positive correlation between receptor level and response to treatment with steroids (Lippman *et al.*, 1978). Furthermore there are indications that the receptor levels in ALL represent a prognostic variable which to some extent is independent of the immunological phenotype. Patients with high receptor content in their leukaemic cells had a long remission contrasting those with low receptor levels who had a shorter remission duration (Lippman *et al.*, 1978).

In the case of AML the receptor level, as determined by *whole cell* assay, does not appear to be related to survival or response to chemotherapy (Bell, 1982). However, in a recent study we reported that there exists a positive correlation between cellular *cytosol* receptor content and survival in AML (Skoog *et al.*, 1982).

The present paper extends our previous study of TdT and glucocorticoid receptor as prognostic indicators in AML.

### Material and methods

### Patients

We have studied 52 adult patients with acute myeloid leukemia (AML). Peripheral blood was collected before therapy was started. All the patients were treated according to the protocol of the Leukaemia Group of Central Sweden (LCS). Thirty patients were less than 60 years old and were treated with daunorubicin and cytarabine initially and if complete remission could be established they were given courses of either daunorubicin and cytarabine or cytarabine and thioguanine as maintenance therapy. Twenty-two patients were 60 years or older, and received a somewhat milder initial treatment with thioguanine and cytarabine. A few elderly patients were treated with prednimustine (LEO, Sweden) as well.

### Criteria for diagnosis. Classification

Our criteria for the diagnosis acute leukaemia were the findings of $\geq 50$ percent leukaemic cells in the bone marrow or an unequivocal finding of Auer rods. The classification was made according to the FAB classification (Bennet *et al.*, 1976) with slight modifications (Ost *et al.*, 1983).

Cytochemical methods used for classification, the procedure for isolation of leukaemic cells from peripheral blood and measurements of TdT activity have been published previously (Skoog *et al.*, 1983).

### Glucocorticoid receptor determinations

Ficoll-Isopaque purified blast cells ($>2 \times 10^7$) were homogenized in 0.5 ml of ET buffer (10 mM Tris-HCl, pH 7.4, 1.5 mM EDTA) containing 10% glycerol (v/v), 1 mM dithiotreitol and 50 nM 1.2 (n) × [$^3$H]-dexamethasone (Amersham 25 Ci nmole$^{-1}$). After homogenization the mixture was incubated at 4°C for 30 min and then centrifuged at 15.000 *g* for 20 min at 4°C. The pellet was used for DNA determination as described by Burton (1968). Unbound steroid was removed by treatment with dextran coated charcoal and trypsinized as outlined elsewhere (Wrang *et al.*, 1981). The receptor – [$^3$H]-dexamethasone complex was isolated by isoelectric focusing in slabs of polyacrylamide gel as prebiously described (Wrang *et al.*, 1981). The receptor – [$^3$H]-dexamethasone complex focused at pH 5.8.

### Immunofluorescent staining of cells in suspension

The following monoclonal antibodies were used for phenotypic characterization of the leukaemic cells: $J_5$ against common ALL antigen and $B_1$ against B-lymphocyte antigen, both kindly provided by Dr Schlossmann. OKla (HLA class II) (Ortho Diagnostics, Sweden), 0KT3 (mature T-lymphocyte antigen) (Ortho Diagnostics, Sweden), $NA_1/34$ (human thymocyte antigen) McMichael, Sera Labs, England), A3/10 (HLA class 1) (Trowbridge) kindly provided by Dr Trowbridge.

Thirty microliters of appropriately diluted (1/1–1/5) antibodies were added to $10^6$ pelleted cells and were incubated for 1 h at 4°C. After washing 3 times, the incubation was repeated with 30 $\mu$l of 1/20 diluted fluorescein isothiocyanate-conjugated *F(ab')*2 sheep anti-mouse Ig serum preabsorbed with human serum (Natl. Bact. Lab., Stockholm, Sweden).

### Immunofluorescent staining of TdT

For immunofluorescent staining (IFL) of TdT, two affinity purified rabbit antisera were used, one was kindly provided by Dr Bollum and the other was purchased from PL Biochemical (S.T. Goar, FRG). The thawed leukaemic cells were Ficoll–Paque separated prior to cytocentrifugation. The slides were air-dried, fixed in cold methanol (30 min at 4°C) and dehydrated in PBS. Five microliters rabbit anti-TdT was added and the slides incubated (30 min at 20°C) in a humidified chamber. The slides were washed in PBS (30 min) before repeating the staining procedure with purified sheep anti-rabbit Ig labelled with FITC (Natl. Bact. Lab., Stockholm, Sweden) for 30 min at 20°C. After repeated washing in PBS (30 min at 20°C), the slides were sealed in formolglycerol under a coverslip and examined in a standard Zeiss microscope with epi-illuminator and 63x oil phase contrast objective.

Only cells which by phase contract were considered as blastic or mononuclear were evaluated, and the frequency of stained nuclei was scored.

### Results

### *Patients*

The mean values of WBC, frequency of Auer rods, percentage of leukemic cells in peripheral blood and the bone marrow in the different FAB subclasses are given in Table I. It can be seen from this table that 29 cases were classified as acute myeloblastic leukaemias (M1-2), 23 of which showed some degree of maturation (M2). The number of monocytic leukaemias (M5a-M5b) was 11, and the number of myelomonocytic leukaemias (M4) was also 11. It can also be seen from this table that Auer rods were found in 33/52 cases which is in good agreement with observations from a consecutive series at Södersjukhuset, Stockholm (Ost *et al.*, 1982). The finding of Auer rods was not correlated to an increased remission or survival rate.

Complete remission was achieved in 22 of the patients (42.3%). The complete remission rate of the patients <60 years old was 56.7% compared to 22.7% for patients over 60 years. The complete remission rate and the survival rate of the two groups differed significantly ($P=0.014$ and $P=0.09$, respectively). There was no significant difference in either complete remission rate or survival time between the FAB subclasses or between leukaemias with or without Auer rods.

### *Terminal deoxynucleoteodyl transpherase*

The activity of TdT in AML cells is generally undetectable or low and high levels of the enzyme have been interpreted to mean a lymphocytic origin of the leukaemic cells. We found measurable activity of TdT (ranging from 0.02 to 7.84 units $\mu g^{-1}$ DNA) in 24 patients with cytochemically verified AML. In the remaining 28 patients no activity could be detected [<0.01 unit $\mu g^{-1}$ DNA). The mean value for all cases was 0.52 units $\mu g^{-1}$ DNA. Figure 1 depicts the logarithmic values for the TdT activity within the different FAB groups, and it can be seen from this figure that the "pure" myeloid leukemias (M1, M2) contained higher levels of enzyme activity than the monocytic subclasses (M5a + M5b).

We have previously shown that a high cellular level ($\geq$0.10 units $\mu g^{-1}$ DNA) of TdT is positively correlated to response to treatment. In accordance with this we found that patients below 60 years with a TdT value equal or above 0.10 unit $\mu g^{-1}$ DNA had a higher complete remission rate (10/12) than patients with values lower than 0.10 unit $\mu g^{-1}$ DNA (7/18) $P=0.016$. This was also true if the M5 cases, which in general have low TdT levels, were excluded. The survival time was 540 days for patients with high TdT values as compared to 120 days for those with low values ($P=0.010$). No correlation was found between age and TdT level. Among the patients who went into complete remission a survival time of 630 days was observed for those with a high TdT content in their blasts as compared to 363 days for those with a low TdT content. This difference is not statistically significant, which is probably explained by the low number of patients in each group.

### *IFL staining of TdT*

Biochemical determinations of TdT give a mean value for a large number of leukaemic cells. It is thus possible that the moderately elevated levels observed in the M1, M2 cases could result from a contamination with *lymphoid* cells. To analyse this we selected four cases of AML with increased levels of TdT as measured biochemically and performed an IFL staining of TdT. One case of myeloblastic

**Table I** FAB-diagnoses, Auer rods, WBC and percentage of leukaemic cells in peripheral blood and bone marrow

| *FAB-diagnoses* | *No* | (%) | *Cases with Auer rods* | *WBC mean value (range) $10^9 l^{-1}$* | *% of leukaemic cells in the peripheral blood mean value (range)* | *% of leukaemic cells in the bone marrow mean value (range)* |
|---|---|---|---|---|---|---|
| $M_1$ | 6 | (11.5) | 2 | 52 (2–128) | 75 (25–95) | 86 (64–98) |
| $M_2$ | 23 | (44.2) | 18 | 83 (13–170) | 80 (54–99) | 83 (60–97) |
| $M_4$ | 11 | (21.2) | 9 | 69 (5–269) | 64 (20–97) | 71 (44–95) |
| $M_5a$ | 3 | (5.8) | 1 | 137 (40–310) | 98 (98–99) | 95 (94–96) |
| $M_5b$ | 8 | (15.4) | 3 | 76 (35–145) | 74 (30–95) | 80 (62–94) |
| $M_6$ | 1 | (1.9) | 0 | 40 | 47 | 55 |
| Total | 52 | (100) | 33 | | | |

| FAB | M1 | M2 | M4 | M5a | M5b | M6 | Total |
|---|---|---|---|---|---|---|---|
| No | 6 | 23 | 11 | 3 | 8 | 1 | 52 |
| Mean value | 1.37 | 0.63 | 0.28 | 0.02 | 0.01 | (0.58) | 0.52 Units |
| Range | 0.01–3.16 | 0.01–7.84 | 0.01–1.24 | 0.01–0.04 | — | — | 0.01–7.84 Units |
| Median value | 0.96 | 0.02 | 0.02 | 0.01 | 0.01 | — | 0.01 Units |

**Figure 1** TdT values in different FAB subclasses.

leukaemia without maturation (M1) showed a positive nuclear staining for TdT in 50% of the cells; the biochemically determined value was 1.75 units $\mu g^{-1}$ DNA. The percentage of cells showing peroxidase positivity was 95%. A case of M2 had a TdT activity of 7.84 units $\mu g^{-1}$ DNA with 62% of peroxidase positive cells and 85% of the cells showed positive nuclear staining for TdT with the immunofluorescense method. Only 5% of the cells were $J_5$-positive and 1% of the cells were positive for 0KT3.

Similar results were observed for two other cases of M1-M2. It thus appears to be clear from these results that the enzyme TdT is found in the leukaemic myeloid cells since the majority of the cells showed positivity both for peroxidase and TdT.

*Glucocorticoid receptor*

We have previously described that AML cells contain varying amounts of the cytoplasmic receptor for glucocorticoids. In the 52 AML cases now tested the glucocorticoid receptor levels ranged from 0 to 2.8 fmol $\mu g^{-1}$ DNA. The content of the glucocorticoid receptor was not significantly correlated to the age of the patients. The mean and median value was 0.31 and 0.22 fmol $\mu g^{-1}$ DNA, respectively. Figure 2 summarizes the glucocorticoid receptor content within the different FAB subclasses and we were unable to detect any differences in glucocorticoid receptor content between the groups. Correlations of the cellular levels of glucocorticoid receptor to remission rate and survival showed that patients (all ages) with a high content of glucocorticoid receptor ($\geq$0.22 fmol $\mu g^{-1}$ DNA) in their leukaemic cells had a complete remission rate of 57% while only 25% of those with a low receptor content ($<$0.22 fmol $\mu g^{-1}$ DNA) responded. This difference is significant with a $P$ value of 0.019. We were unable to find such a difference between the receptor rich and poor groups if patients under or over 60 years were analyzed separately. Moreover no statistically significant differences was observed concerning time of survival.

*Prognostic significance of combined glucocorticoid receptor levels and TdT values*

The cellular level of TdT and the glucocorticoid receptor were not correlated with each other ($P = 0.30$). Since both parameters were shown to give prognostic information it was of interest to analyse if their usefulness as predictors of prognosis could be increased if they were combined.

Among the 52 patients studied 10 had both an increased glucocorticoid receptor content ($\geq$0.22 fmol $\mu g^{-1}$ DNA) and TdT activity ($\geq$0.1 unit $\mu g^{-1}$ DNA. Nine of these patients went into complete remission upon therapy. In the group of 16 patients with receptor poor and TdT poor cells only 4 (25%) achieved complete remission. These remissions were all confined to M5 cases. Of the remaining 12 patients 8 survived for more than 30 days and were given an adequate therapeutic trials.

| FAB | M1 | M2 | M4 | M5a | M5b | M6 | Total |
|---|---|---|---|---|---|---|---|
| No | 6 | 23 | 11 | 3 | 8 | 1 | 52 Units |
| Mean value | 0.28 | 0.38 | 0.24 | 0.41 | 0.22 | (0.08) | 0.31 Units |
| Median value | 0.32 | 0.29 | 0.15 | 0.32 | 0.13 | | 0.22 Units |

**Figure 2** Glucocorticoid receptor values in different FAB subclasses.

The difference in complete remission rate is statistically significant ($P=0.001$). There was also a significant difference ($P=0.007$) between the two groups concerning survival as shown in Figure 3. This difference was also found when patients under 60 years were studied ($P=0.007$). There were only 2 patients over 60 years who had high levels of both TdT and the glucocorticoid receptor. One of these patients survived for 759 days while the other one lived for 2 days. Seven patients over 60 years showed low values for both the receptor and TdT. Only 2 of these patients survived for more than 120 days (239 and 157 days respectively). We thus conclude that a combination of the glucocorticoid receptor level and TdT content increase their usefulness as predictors of prognosis.

**Figure 3** The survival time of patients with a high content of TdT ($\geq 0.1$ unit $\mu g^{-1}$ DNA) and the glucocorticoid receptor ($\geq 0.22$ fmole $\mu g^{-1}$ DNA) as compared to that of patients with a low level of TdT ($<0.1$ unit $\mu g^{-1}$ DNA) and the receptor ($<0.22$ fmol $\mu g^{-1}$ DNA). The unbroken line represents patients with a high TdT and receptor content and the dotted line those with a low enzyme and receptor level.

### Discussion

The material we present in this paper represents a group of AML cases only. All patients with a dysmyeloplastic syndrome before diagnosis, ALL (according to ummunological markers) and one case of mixed leukaemia (ALL+M4) were excluded. We are thus convinced that the patients included in the present study are "pure" AML. cases. It is therefore of interest that 24 of these "pure" AML had measurable activity of TdT in their leukaemic cells. It should be noted that the level of TdT was considerably lower (1/10–1/100) than that observed in ALL cells and cells from some blast crisis of chronic myeloid leukaemias. Immunofluorescence staining for TdT presented two important results. Firstly, the enzyme occurs in the nucleus of myeloid cells. Secondly, it appears that the IFL positivity is evenly distributed among a fraction of the cells. These findings conclusively show that a varying proportion of the leukaemic cells in some myeloid leukaemias contain TdT, albeit at a lower level than lymphoid cells.

There are several previous reports on the prognostic value of TdT determinations in leukaemia Skoog *et al.*, 1982; Marks *et al.*, 1978; Mertlesman, 1982; Sasaki *et al.*, 1981). The majority of these studies have been on cells from patients with ALL and CML in blast crisis. In such cases TdT positivity indicates a high rate of responsiveness to chemotherapy Marks *et al.*, 1978; Sasaki *et al.*, 1981). In an earlier study of AML we found that patients whose leukaemic cells have an increased level of TdT have both higher remission and survival rates (Skoog *et al.*, 1982). The number of patients was, however, small and it was therefore of interest to confirm our findings on a larger group of patients.

The present article confirms that AML patients (<60 years) with high cellular levels of the enzyme have a significantly higher remission rate than those with low levels. These results are in contrast to those reported by Mertelsman (1982) who found that increased levels of TdT was correlated to a low remission rate and a shorter survival. In the latter study TdT positivity was defined in a semiquantitative way whereas our results are based on absolute values. This may partly explain the discrepancies observed although the biochemical background remains to be explored.

Mertelsman (1982) also described that the presence of Auer rods in TdT negative patients was associated with an increased remission rate. In our material we were unable to attach any prognostic information to the finding of Auer rods.

There are several reports on the presence of glucocorticoid receptors in normal and neoplastic lymphoid and myeloid cells as well as in erythropoietic cells. In ALL high receptor levels appeared to be a prognostic favourable sign (Lippman *et al.*, 1978; Sasaki *et al.*, 1981). In the case of AML conflicting results have been presented. Bell (1982) who measured the receptor in intact cells could not find any correlation between prognosis and receptor levels. In contract to this we have previously shown that a high level of the glucocorticoid receptor, when measured in the cellular cytosol, was positively correlated to the remission rate as well as survival. The present study shows that patients having a receptor level equal or above 0.22 fmol $\mu g^{-1}$ DNA have a significantly higher complete remission rate than patients with a low receptor value. However, we could not observe any difference in length of survival or length of first complete remission between the receptor rich and receptor poor groups in this material. At present we are unable to explain the discrepancies beween the results obtained with *whole cell* measurements and those based on *cytosol* receptor determinations. It could be speculated that the receptor values as determined by the whole cell technique depend not only on the amount of free cytosol receptor but also on the rate of translocation of the steroid-receptor complex into the nucleus and rate of degradation. No translocation or degradation occurred with the technique for receptor determination used in this paper.

Unfortunately there are no antibodies available to the glucocorticoid receptor and it is thus not possible to show conclusively that the receptor as measured biochemically is located in all the leukaemic cells. When such antibodies are available it will be interesting to study the inter-and intracellular distribution in the leukaemic cells.

The levels of TdT and the glucocorticoid receptor may represent separate prognostic parameters. Thus, by combining both variables it was possible to show that patients with high levels of both TdT and the receptor had significantly higher remission rate and longer survival time as compared to those with low values of TdT and the receptor. A similar finding has been reported for patients with ALL and CML in blast crisis (Sasaki *et al.*, 1981). It is of interest to note that the remission rate and survival time did not correlate to other variables such as WBC, FAB subclass and the finding of Auer rods in our material. Although it is hard to explain the basis for prognostic prediction using TdT and the glucocorticoid receptor it is of importance to report such results since there are few good prognostic indicators in AML. It is possible that determinations of TdT and the glucocorticoid receptor can be of importance when planning new therapeutic strategies in the future. Furthermore it seems questionable if patients above 60 years of age with low TdT and low glucocorticoid receptor levels have any benefit from cytostatic drugs in the combinations and doses used in this series.

The results presented in this paper further stress the great heterogeneity of the AML which is a challenging field for future research.

### References

BELL, P.A. (1982). Glucocorticoids in the therapy of leukemia and lymphoma. *Clin. Oncol.*, **1**, 131.

BENNET, J.M., CATOVSKY, D., & DANIEL, M.T. & 0 others (1976). Proposals for the classification of the acute leukeamias. *Br. J. Haematol.*, **33**, 451.

BURTON, K. (1968). Determination of DNA concentration with diphenylamine. In *Methods in Enzymology* (Eds. Grossman & Moldave) p. 163, Vol. XII, part B. Academic Press, New York.

CHILDREN'S CANCER STUDY GROUP (1967). Prednisone therapy of acute childhood leukemia: prognosis and duration of response in 330 patients. *J. Pediatr.*, **70**, 626.

HOFFBRAND, A., GANESHAGURU, K., JANOSSY, G., GREAVES, M.F., CATOVSKY, D. & WOODRUFF, R.K. (1977). Terminal deoxynucleotidyl-transferase levels and membrane phenotypes in diagnosis of acute leukaemia. *Lancet* **ii**, 520.

KEATING, M.J., SMITH, T.L., GEHAN, E.R. & 6 others (1980). Factors related to length of complete remission in adult acute leukaemia. *Cancer*, **45**, 2017.

KNOSPE, W.H. & CONRAD, M.E. (1966). The danger of corticosteroids in acute granulocytic leukemia. *Med. Clin. N. Am.*, **50**, 1653.

LIPPMAN, M.E., YARBRO, G.K. & LEVENTHAL, B.G. (1978). Clinical implications of glucocorticod receptors in human leukemia. *Cancer Res.*, **38**, 4251.

MARCUS, S.L., SMITH, S.W., JAROWSKI, C.I. & MODAK, M.J. (1976). Terminal deoxyribonucleotidyl transferase activity in acute undifferentiated leukaemia. *Biochem Biophys Res Commun.*, **70**, 37.

MARKS, M.S., BOLTIMORE, D. & McCAFFREY, R. (1978). Terminal transferase as a predictor of initial responsiveness to vincristine and prednisone in blastic chronic myelogenous leukaemia. *N. Engl. J. Med.*, **298**, 812.

MERTELSMAN, R. (1982). The prognostic significance of terminal deoxynucleotidyl transferase (TdT) in patients with leukemias and malignant lymphomas. In: *Terminal Transferase in Immunobiology and Leukemia.* (Eds. Bertazzoni & Bollum) Plenum.

ÖST, Å., LAGERLÖF, B., SUNDSTRÖM, C. & 4 others (1983). A study of the reproducibility of the diagnostic criteria for acute leukemia. *Scand J. Hematol.*, **31**, 257.

SASAKI, R., TAKAKU, F., AOKI, T., BOLLUM, F.J., SAITO, T. & DAN, S. (1981). Terminal deoxynucleotidyl transferase activities and glucocorticoid receptors in leukemia. *Br. J. Cancer*, **44**, 63.

SHANBROM, E. & MILLER, S. (1962). Critical evaluation of massive steroid therapy of acute leukaemia. *N. Engl. J. Med.*, **266**, 1354.

SKOOG, L., NORDENSKJÖLD, B., ÖST, Å., & 6 others (1982). Glucocorticoid receptor concentrations and terminal transferase activity as indicators of prognosis in acute non-lymphocytic leukaemia. *Br. Med. J.*, **282**, 1826.

SRIVASTAVA, B.I.S., KHAN, S.A. & HENDERSSON, E.S. (1976). High terminal deoxynucleotidyl transferase activity in acute myelogenous leukemia. *Cancer Res.*, **36**, 3847.

WRANGE, Ö., HUMLA, S., RAMBERG, J. & 4 others (1981). Progestin-receptor analysis in human breast cancer cytosol by isoelectric focusing in stabs of polyacrylamide gel. *J. Steroid Biochem.*, **14**, 141.